# Supplementary figures and images for: Two-years mothering into the pandemic: Impact of the three COVID-19 waves in the Argentinian postpartum women’s mental health
Source: PLoS One. 2025 Mar 19;20(3):e0294220. doi: 10.1371/journal.pone.0294220 (PMC11922242; doi:10.1371/journal.pone.0294220)

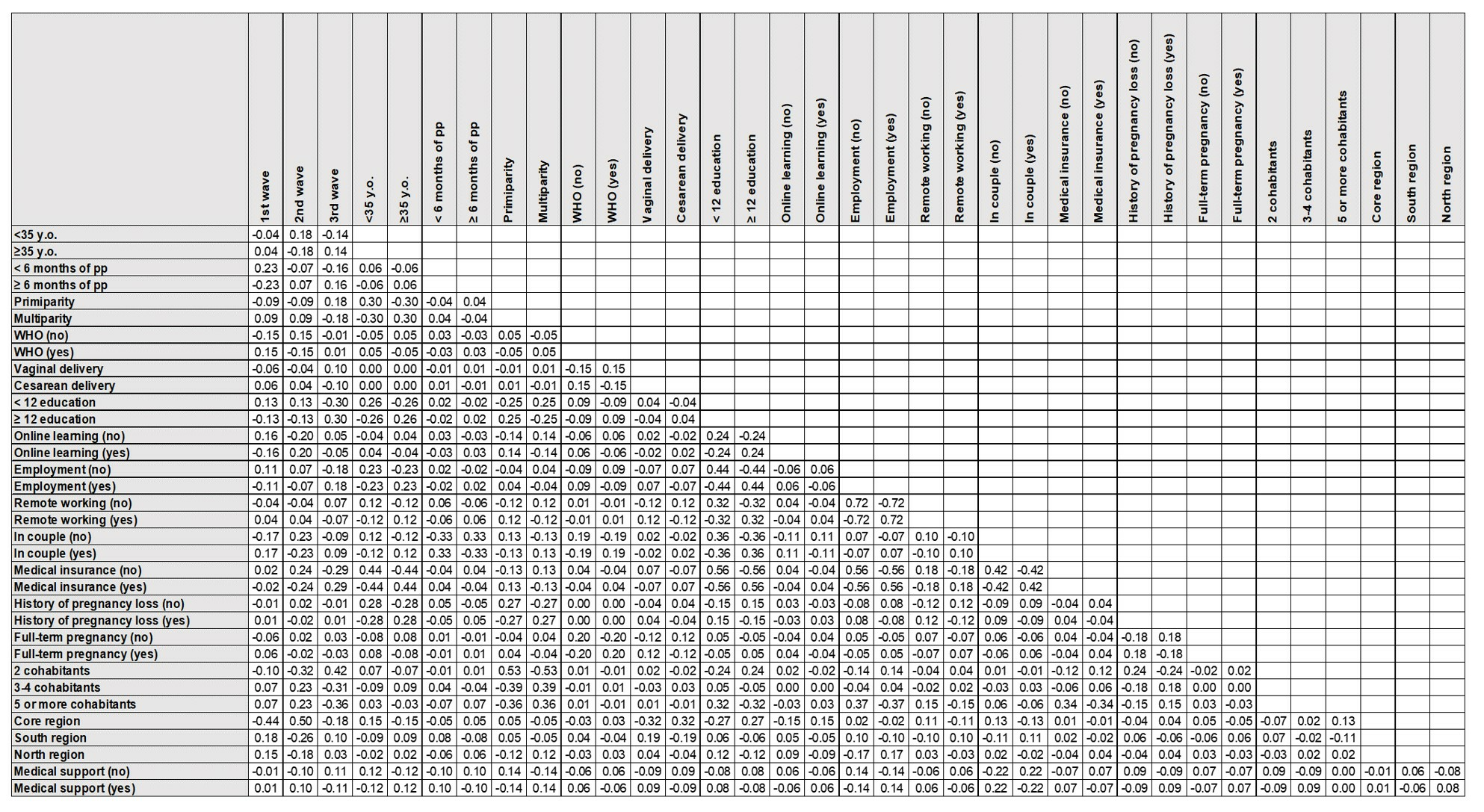

Supplement: S1 Fig — (TIF) [file pone.0294220.s001.tif]

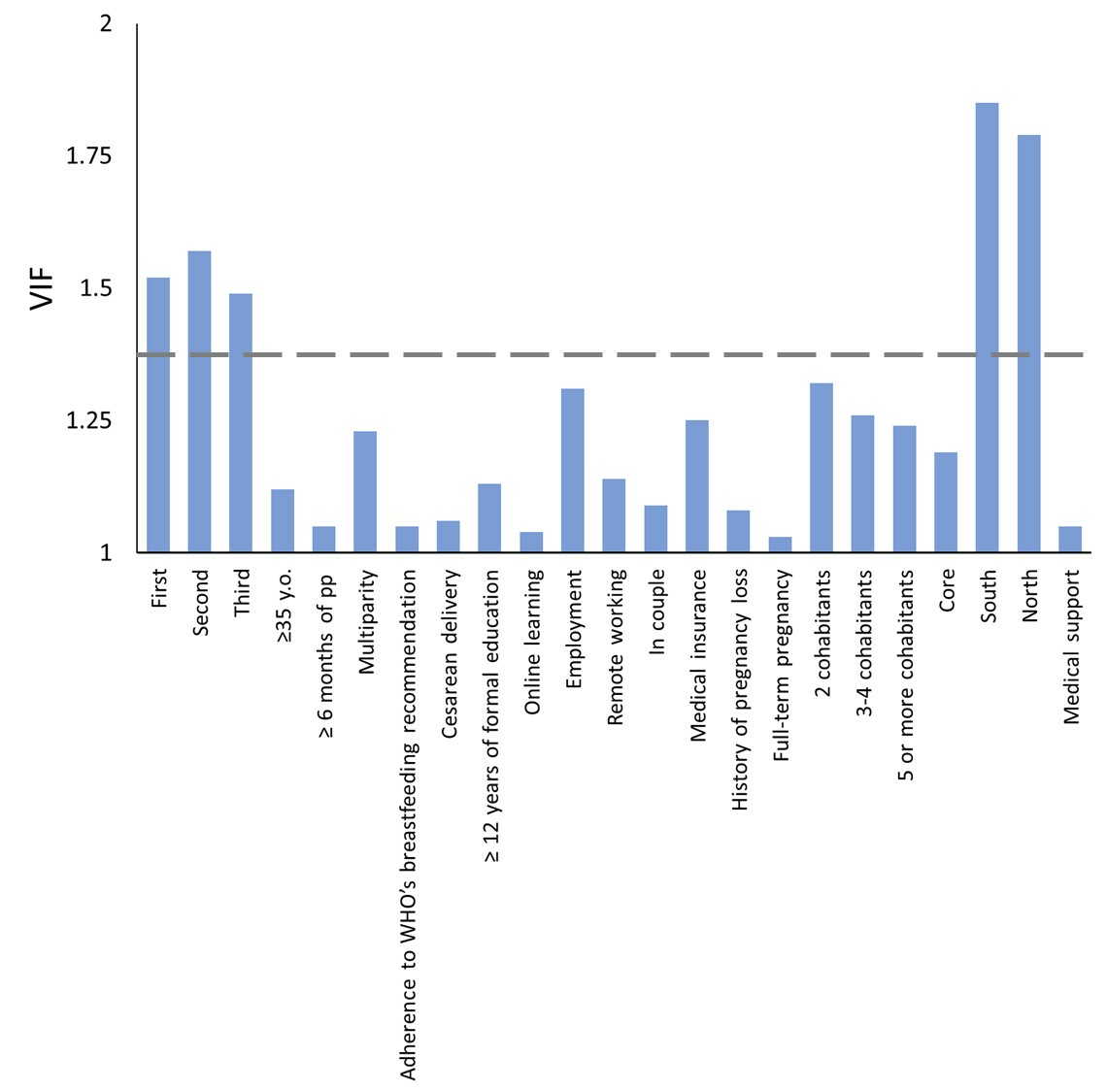

Supplement: S2 Fig — (TIF) [file pone.0294220.s002.tif]
