# Supplementary material for: Two-years mothering into the pandemic: Impact of the three COVID-19 waves in the Argentinian postpartum women’s mental health
Source: PLoS One. 2025 Mar 19;20(3):e0294220. doi: 10.1371/journal.pone.0294220 (PMC11922242; doi:10.1371/journal.pone.0294220)
Supplement: Attachment — Submitted filename: Response to Reviewers.docx [file pone.0294220.s003.docx]

Dear Editor,

We appreciate the valuable suggestions provided by the reviewers and yourself, which have greatly enhanced our work. Consequently, each change made is indicated in the following list:

**Editor’s comments:**

1. Please ensure that your manuscript meets PLOS ONE's style requirements, including those for file naming.

**Response: The manuscript was formatted following the indications in the template provided by the Editor.**

2. You indicated that ethical approval was not necessary for your study. We understand that the framework for ethical oversight requirements for studies of this type may differ depending on the setting and we would appreciate some further clarification regarding your research. Could you please provide further details on why your study is exempt from the need for approval and confirmation from your institutional review board or research ethics committee (e.g., in the form of a letter or email correspondence) that ethics review was not necessary for this study? Please include a copy of the correspondence as an ""Other"" file.

**Response: As described in the Materials and Methods section, this study had the corresponding ethical approval. “This research was approved by the corresponding Research Ethics Committee (registration codes REPIS-3177; REPIS-011), following the Declaration of Helsinki and current legislation”.**

3. Note from Emily Chenette, Editor in Chief of PLOS ONE, and Iain Hrynaszkiewicz, Director of Open Research Solutions at PLOS: Did you know that depositing data in a repository is associated with up to a 25% citation advantage (https://doi.org/10.1371/journal.pone.0230416)? If you’ve not already done so, consider depositing your raw data in a repository to ensure your work is read, appreciated and cited by the largest possible audience. You’ll also earn an Accessible Data icon on your published paper if you deposit your data in any participating repository (https://plos.org/open-science/open-data/#accessible-data).

**Response: The data presented in this study are openly available in Open Science Framework (OSF) at** [**https://osf.io/v69tf/**](https://osf.io/v69tf/)

4. Thank you for stating the following financial disclosure: 'EAS. Secretaría de Ciencia y Tecnología, Universidad Nacional de Córdoba (grant number SECYT-UNC 273/2020)."

Please state what role the funders took in the study. If the funders had no role, please state: ""The funders had no role in study design, data collection and analysis, decision to publish, or preparation of the manuscript.""

If this statement is not correct you must amend it as needed.

Please include this amended Role of Funder statement in your cover letter; we will change the online submission form on your behalf.

**Response: The following statement was added "The funders had no role in study design, data collection and analysis, decision to publish, or preparation of the manuscript."**

5. In the online submission form, you indicated that data contain private health information and cannot be shared publicly. The minimal dataset will be made available upon request for researchers who meet the criteria for access to confidential data. Interested researchers should contact the corresponding author on agustin.miranda@ird.fr

All PLOS journals now require all data underlying the findings described in their manuscript to be freely available to other researchers, either 1. In a public repository, 2. Within the manuscript itself, or 3. Uploaded as supplementary information.

This policy applies to all data except where public deposition would breach compliance with the protocol approved by your research ethics board. If your data cannot be made publicly available for ethical or legal reasons (e.g., public availability would compromise patient privacy), please explain your reasons on resubmission and your exemption request will be escalated for approval.

**Response: The data presented in this study are openly available in Open Science Framework (OSF) at** [**https://osf.io/v69tf/**](https://osf.io/v69tf/)

6. We note that Figure 2 in your submission contain [map/satellite] images which may be copyrighted. All PLOS content is published under the Creative Commons Attribution License (CC BY 4.0), which means that the manuscript, images, and Supporting Information files will be freely available online, and any third party is permitted to access, download, copy, distribute, and use these materials in any way, even commercially, with proper attribution. For these reasons, we cannot publish previously copyrighted maps or satellite images created using proprietary data, such as Google software (Google Maps, Street View, and Earth). For more information, see our copyright guidelines: http://journals.plos.org/plosone/s/licenses-and-copyright.

We require you to either (1) present written permission from the copyright holder to publish these figures specifically under the CC BY 4.0 license, or (2) remove the figures from your submission.

**Response: The map was enhanced and made in Stata using open license data. We include the following statement: “This map was created with the spmap package in Stata 18 by the authors, using shapefiles from the open license resource geoBoundaries Global Administrative Database (**[**www.geoboundaries.org**](http://www.geoboundaries.org)**) [25].”**

7. Please include captions for your Supporting Information files at the end of your manuscript, and update any in-text citations to match accordingly. Please see our Supporting Information guidelines for more information: http://journals.plos.org/plosone/s/supporting-information.

**Response: Done.**

8. Please review your reference list to ensure that it is complete and correct. If you have cited papers that have been retracted, please include the rationale for doing so in the manuscript text, or remove these references and replace them with relevant current references. Any changes to the reference list should be mentioned in the rebuttal letter that accompanies your revised manuscript. If you need to cite a retracted article, indicate the article’s retracted status in the References list and also include a citation and full reference for the retraction notice.

**Response: We confirmed that there were no retractions among the references used in the current study. Only one reference (Rogowska et al., 2021) had a corrigendum issued after publication, which corrected an error in the figure captions. This correction did not affect the study's conclusions, and the main document has been updated accordingly.**

**Additional Editor Comments:**

1. **Comment:** How do you take care of participants who respond to the questionnaire twice or more taking into consideration that it took about two years to gather the data (3 waves). **Response:** **We added the following rationale in Materials and methods:**

*“Some measures were conducted to avoid repeated responses. In this sense, the time-limited nature of puerperium and the absence of any compensation for participating discouraged multiple responses by the same participants. Additionally, a unique identifier was requested before accessing the questionnaire, consisting of the combination of the first two initials of the first and last name and birth date. This identifier served to control the response reliability and potential repetitions. In order to protect sensitive personal information that could compromise anonymity given ethical constraints, no further data about participants’ identity was collected.”*

2. **Comment:** What are the limitations to using data of three waves spanning over two years especially as all the data were combined too (n=1000):

**The following rationale was included in Discussion:**

*“Despite that, interpretations derived from the aggregation of data from different waves may be affected by the social context and specific characteristics of each of the three subsamples. These factors included events such as the changes in health policies related to the pandemic over the study duration [82]. Nonetheless, the multivariate statistical modeling performed herein considers the pandemic waves as potential confounders to obtain reliable and precise estimates. Also, it is necessary to emphasize that, although repeated cross-sectional studies are inappropriate to observe individual-level changes, they are adequate to assess population-level changes [83].”*

**Reviewer #1:** Thank you very much for the possibility to review the study titled "Two-years mothering into the pandemic: Impact of the three COVID-19 waves in the Argentinian postpartum women’s mental health".

The paper is very interesting and well written. There are only small points to review that could improve the work. In particular, although the introduction and discussions address the issue of the pandemic and the impact it can have very well,

1. Comment: It would be important to be able to better discuss these aspects with the difficulties that women experience on an intrapsychic level in the transition to motherhood. In particular, it is suggested to see some studies in this regard:

- Tambelli, R., Ballarotto, G., Trumello, C., & Babore, A. (2022). Transition to Motherhood: A Study on the Association between Somatic Symptoms during Pregnancy and Post-Partum Anxiety and Depression Symptoms. International Journal of Environmental Research and Public Health, 19(19), 12861.

- Ben-Ari, O. T., Shlomo, S. B., Sivan, E., & Dolizki, M. (2009). The transition to motherhood—A time for growth. Journal of Social and Clinical Psychology, 28(8), 943-970.

- Rallis, S., Skouteris, H., McCabe, M., & Milgrom, J. (2014). The transition to motherhood: towards a broader understanding of perinatal distress. Women and Birth, 27(1), 68-71.

Furthermore, the paper partly takes for granted the importance of assessment and prevention in this specific age group. Although there is a very large literature that has highlighted the outcomes in children with mothers suffering from post-partum depression, I think it is important to highlight it, both in the introduction and more importantly in the conclusions and clinical implications. For example, several studies have highlighted the effects both in early childhood and later in children, highlighting the importance of interventions. For example:

- Ballarotto, G., Murray, L., Bozicevic, L., Marzilli, E., Cerniglia, L., Cimino, S., & Tambelli, R. (2023). Parental sensitivity to toddler’s need for autonomy: An empirical study on mother-toddler and father-toddler interactions during feeding and play. Infant Behavior and Development, 73, 101892.

- Liu, X., Wang, S., & Wang, G. (2022). Prevalence and risk factors of postpartum depression in women: a systematic review and meta‐analysis. Journal of Clinical Nursing, 31(19-20), 2665-2677.

- Hutchens, B. F., & Kearney, J. (2020). Risk factors for postpartum depression: an umbrella review. Journal of midwifery & women's health, 65(1), 96-108.

- Cimino, S., Cerniglia, L., Tambelli, R., Ballarotto, G., Erriu, M., Paciello, M., ... & Koren-Karie, N. (2020). Dialogues about emotional events between mothers with anxiety, depression, anorexia nervosa, and no diagnosis and their children. Parenting, 20(1), 69-82.

**Response: The authors are grateful for the suggestion of R1. We agree that it is important to address intrapsychic changes related to the transition to motherhood, as well as the impact of mental health issues on child health and development. Accordingly, we have included these aspects in the manuscript based on the literature suggested by the reviewer.**

**In Introduction:**

“*Women face several intrapsychic challenges during the transition to motherhood, characterized by a "new psychic organization" where they redefine their roles and self-perceptions, often experiencing physical and psychological symptoms [5]. These symptoms linked to postpartum depression and anxiety indicate difficulties in adapting to the new roles [5]. Maternal distress-inducing factors also include changes in body, responsibilities, and social circumstances [6, 7]. Effective adaptation to motherhood involves internal resources (e.g., self-esteem and coping strategies) and external resources (e.g., social support), which mitigate this burden and protect mental health [7]. Notably, maternal mental health issues also affect their children. In this sense, when caregiver responsiveness is impaired, maternal-child interactions and attachment are disrupted [8, 9]. Consequently, children may exhibit poorer social engagement, regulatory behaviors, coping, emotionality, sleep quality, eating behavior, and development [10, 11]. Furthermore, long-term outcomes include an increased risk of psychosocial issues in adulthood [11]. Therefore, interventions to address maternal psychological disorders are crucial, as they improve both women and child health, mitigate long-term societal costs, and foster healthier emotional and developmental trajectories for children.*”

**In Conclusion:**

*“Research shows an association between maternal mental health issues and impaired child development, with both short- and long-term outcomes. Thus, our findings emphasize the critical importance of routine mental health assessments during perinatal visits, especially in crisis contexts. This highlights the need for targeted treatments and interventions for women at risk of postpartum depression, stress, and insomnia.”*

2. Comment: Abstract: Acronyms for tools are used in the abstract. Authors are invited to eliminate acronyms. Specifically: "Postpartum depression (PDSS‐SF), insomnia (ISI), and perceived stress symptoms27 (PSS‐C) were used"

**Response: Done.**

3. Comment: Finally, the authors are invited to better clarify the hypotheses of the study, justifying them with the literature.

**Response: The hypothesis of the study was included in the Introduction section.**

**Reviewer #2:** The authors have done a good job with the organization of their article in general. To begin with, the research has been dealt with the technical aspect very well, and the conclusion has a very strong link that makes the article a very good one. In addition, the statistical aspect of the article is presented in a professional manner, although I have not gotten access to the original data to examine it. Furthermore, the authors have indicated that the data are available without restriction. Finally, the article presents the write-up in professional and appropriate English grammar throughout the article. I do recommend this article without any competing interest for publication.

**Response: We greatly appreciate R2's comments. The data presented in this study are now openly available in Open Science Framework (OSF) at https://osf.io/v69tf/**

**General changes:**

**References: The following additional references were included to comply with the reviewers' comments:**

5.	Tambelli R, Ballarotto G, Trumello C, Babore A. Transition to Motherhood: A Study on the Association between Somatic Symptoms during Pregnancy and Post-Partum Anxiety and Depression Symptoms. Int J Environ Res Public Health. 2022;19(19):12861. doi: 10.3390/ijerph191912861

6.	Rallis S, Skouteris H, McCabe M, Milgrom J. The transition to motherhood: towards a broader understanding of perinatal distress. Women Birth. 2014;27(1):68-71. doi: 10.1016/j.wombi.2013.12.004

7.	Ben-Ari OT, Shlomo SB, Sivan E, Dolizki M. The transition to motherhood—A time for growth. Journal of Social and Clinical Psychology. 2009;28(8):943-70. doi: 10.1521/jscp.2009.28.8.943

8.	Cimino S, Cerniglia L, Tambelli R, Ballarotto G, Erriu M, Paciello M, Oppenheim D, Koren-Karie N. Dialogues about emotional events between mothers with anxiety, depression, anorexia nervosa, and no diagnosis and their children. Parenting. 2020;20(1):69-82. doi: 10.1080/15295192.2019.1642688

9.	Ballarotto G, Murray L, Bozicevic L, Marzilli E, Cerniglia L, Cimino S, Tambelli R. Parental sensitivity to toddler's need for autonomy: An empirical study on mother-toddler and father-toddler interactions during feeding and play. Infant Behav Dev. 2023;73:101892. doi: 10.1016/j.infbeh.2023.101892

10.	Liu X, Wang S, Wang G. Prevalence and Risk Factors of Postpartum Depression in Women: A Systematic Review and Meta-analysis. J Clin Nurs. 2022;31(19-20):2665-2677. doi: 10.1111/jocn.16121

11.	Hutchens BF, Kearney J. Risk Factors for Postpartum Depression: An Umbrella Review. J Midwifery Womens Health. 2020;65(1):96-108. doi: 10.1111/jmwh.13067

25.	Runfola D, Anderson A, Baier H, Crittenden M, Dowker E, Fuhrig S, et al. geoBoundaries: A global database of political administrative boundaries. PLoS One. 2020;15(4):e0231866. doi: 10.1371/journal.pone.0231866

82.	Rafferty A, Walthery P, King-Hele S (2015). “Analysing change over time: repeated cross-sectional and longitudinal survey data”, UK Data Service, University of Essex and Manchester, available at: https://dam.ukdataservice.ac.uk/media/455362/changeovertime.pdf (accessed 12 June 2024).

83.	Maletta RM, Daly M, Goodwin L, Noonan R, Putra IGNE, Robinson E. Changes in the prevalence of perceived discrimination and associations with probable mental health problems in the UK from 2015 to 2020: A repeated cross-sectional analysis of the UK Household Longitudinal Study. SSM Popul Health. 2024;26:101667. doi: 10.1016/j.ssmph.2024.101667.

In addition, the year of publication of the following reference was updated:

75. King LS, Feddoes DE, Kirshenbaum JS, Humphreys KL, Gotlib IH. Pregnancy during the pandemic: the impact of COVID-19-related stress on risk for prenatal depression. Psychol Med. 2023;53(1):170-180. doi: 10.1017/S003329172100132X

All issues raised by the editor and reviewers have been addressed. Thus, the authors hope that this article will be appropriate for Plos One.

Respectfully,

**Agustin Ramiro Miranda**

Institut de Recherche pour le Développement (IRD)
