# Supplementary material for: Two-years mothering into the pandemic: Impact of the three COVID-19 waves in the Argentinian postpartum women’s mental health
Source: PLoS One. 2025 Mar 19;20(3):e0294220. doi: 10.1371/journal.pone.0294220 (PMC11922242; doi:10.1371/journal.pone.0294220)
Supplement: Attachment — Submitted filename: Response to reviewer.docx [file pone.0294220.s005.docx]

Dear Editor,

We thank the reviewer for the insightful suggestions, which have greatly improved our work. The changes made are listed below:

- **Comment:** The abstract should adjusted for clarity especially on the concerned factors, such can be named negative factors affecting mental health.(line 31)

**Response:** The sentence has been rephrased for clarity.

- **Comment:** ‘No medical support’ can be changed to ‘lack of medical support’ (line 31)

**Response:** ‘No medical support’ was replaced by ‘lack of medical support’.

- **Comment:** Line 38 to 40 in abstract can be rephrased to “*Thus, health systems must see to protection of women of reproductive age group against negative factors in order to cope with pandemic-related events*”.

**Response:** The sentence has been rephrased.

- **Comment:** The introduction can be improved by mentioning pre-Covid 19 data on postpartum depression briefly to expatiate on the disease burden.

**Response:** This issue has been addressed in the Discussion section (paragraph 3, lines 381-383). Also, the pre-COVID prevalence of maternal mental health issues has been addressed in the Introduction section (paragraph 3, lines 76-79).

- **Comment:** Methodology is adequate. Type of lactation should be changed to type of feeding since mixed feeding and artificial formula were part of the options.

**Response:** “Type of lactation” has been replaced by “type of feeding”.

- **Comment:** Conclusion can be trimmed down to few details on the findings and recommendations against future similar epidemiologic scenarios.

**Response:** Conclusion section has been revised and re-written to focus more concisely on the key findings and recommendations for future similar epidemiologic scenarios, as follows: “*In summary, our results demonstrate that the mental health of Argentinian puerperal women deteriorated during the COVID-19 pandemic. Our research findings suggest a notable increase in the prevalence of postpartum depression and insomnia during the outbreaks. Moreover, the social and economic impact involved a high psychological risk, although women have become able to cope with pandemic-related stress. Thus, these findings emphasize the critical importance of routine mental health assessments during perinatal visits, especially in future similar epidemiologic scenarios, to address the risk of postpartum depression, stress, and insomnia for vulnerable mothers*.”

The authors hope that this revised version meets the standards for publication in PLOS ONE.

Respectfully,

Agustin Ramiro Miranda

Institut de Recherche pour le Développement (IRD)
