# Supplementary material for: Two-years mothering into the pandemic: Impact of the three COVID-19 waves in the Argentinian postpartum women’s mental health
Source: PLoS One. 2025 Mar 19;20(3):e0294220. doi: 10.1371/journal.pone.0294220 (PMC11922242; doi:10.1371/journal.pone.0294220)
Supplement: Attachment — Submitted filename: Response.docx [file pone.0294220.s006.docx]

Dear Editor,

We appreciate the valuable suggestions provided by the reviewer, which have enhanced our work. Consequently, each change made is indicated in the following list:

**Comment 1:** Have the authors made all data underlying the findings in their manuscript fully available? Reviewer #4: No

**Response:** As addressed in a previous round, the data presented in this study are openly available in Open Science Framework (OSF) at <https://osf.io/v69tf/files/osfstorage>

**Comment 2:** The manuscript does not address the comments I had made in the previous round of review. I have copied them here: This is an excellent study and a well written paper.

I have queries concerning PSS-C scores:

- PSS-C scores have not been presented for the first wave of the pandemic. Is there any specific reason for this lack of data? This should be mentioned in the methodology.

**Response:** Thank you for your feedback and for bringing this to our attention. We sincerely apologize for the oversight and any confusion caused. The comments from the previous round of review were inadvertently not addressed in the revised manuscript. This was an unintentional error, and we regret that it was missed. We have now carefully reviewed your previous comments and have made the necessary revisions to ensure that all of your suggestions are properly incorporated. The updated manuscript reflects these changes, and we believe they improve the overall quality of the study.

The specific reason for the lack of PSS-C data during the first wave of COVID-19 is that the validation of the instrument was published after this period. This is understandable, as the authors who adapted the PSS to the pandemic context conducted their research at the beginning of the pandemic, with their manuscript being published in July 2020, the same month when data collection for the first sample in our study was completed. This has been addressed in Discussion section as stated in the next response.

- As studying evolution of pandemic related stress was part of the test hypothesis, how did the authors deal with this missing data in their analysis? What possible effect could it have on the results, and conclusions?

**Response:** The following text has been added in Discussion section: “Second, as part of our exploration of the evolution of pandemic-related stress, the absence of PSS-C data during the first wave of COVID-19 represents a potential limitation. This gap arose because the Spanish PSS-C adaptation for the pandemic context was published after data collection for our initial sample had been completed [84]. Furthermore, since the lack of data affected all participants in this wave, rather than a specific subset, we opted not to impute the missing values in order to prevent potential bias. Considering the exceptional circumstances of the first wave, which was marked by unprecedented levels of uncertainty and stress for all, and recognizing that this phase tended to be the most stressful [85], the absence of data during this period is not expected to substantially affect the overall conclusions of the study.”.

**General changes:**

The following additional references were included to comply with the reviewer's comments:

84.	Campo-Arias A, Pedrozo-Cortés MJ, Pedrozo-Pupo JC. Pandemic-Related Perceived Stress Scale of COVID-19: An exploration of online psychometric performance. Rev Colomb Psiquiatr. 2020;49:229-230. English, Spanish. doi: 10.1016/j.rcp.2020.05.005.

85.	Bendau A, Asselmann E, Plag J, Petzold MB, Ströhle A. 1.5 years pandemic - Psychological burden over the course of the COVID-19 pandemic in Germany: A nine-wave longitudinal community study. J Affect Disord. 2022;319:381-387. doi: 10.1016/j.jad.2022.09.

All issues raised by the reviewer have been addressed. Thus, the authors hope that this article will be appropriate for Plos One.

Respectfully,

**Agustin Ramiro Miranda**

Institut de Recherche pour le Développement (IRD)
