# Supplementary material for: Two-years mothering into the pandemic: Impact of the three COVID-19 waves in the Argentinian postpartum women’s mental health
Source: PLoS One. 2025 Mar 19;20(3):e0294220. doi: 10.1371/journal.pone.0294220 (PMC11922242; doi:10.1371/journal.pone.0294220)
Supplement: Attachment — Submitted filename: Reviewer Comment Plos one.docx [file pone.0294220.s004.docx]

Thank you for the call to review the journal titled: ‘’ Two-years mothering into the pandemic: Impact of the three COVID-19 waves in the Argentinian postpartum women’s mental health"

The abstract should adjusted for clarity especially on the concerned factors, such can be named negative factors affecting mental health.(line 31)

‘No medical support’ can be changed to ‘lack of medical support’ (line 32)

Line 38 to 40 in abstract can be rephrased to ‘thus health systems must see to protection of women in reproductive age group against negative factors in order to cope with pandemic related events’

The introduction can be improved by mentioning pre-Covid 19 data on postpartum depression briefly to expatiate on the disease burden.

Methodology is adequate. Type of lactation should be changed to type of feeding since mixed feeding and artificial formula were part of the options.

Conclusion can be trimmed down to few details on the findings and recommendations against future similar epidemiologic scenarios.
